# Successful displacement of a traumatic submacular hemorrhage in a 13-year-old boy treated by vitrectomy, subretinal injection of tissue plasminogen activator and intravitreal air tamponade: a case report

**DOI:** 10.1186/s12886-015-0090-3

**Published:** 2015-08-07

**Authors:** Shinichiro Doi, Shuhei Kimura, Yuki Morizane, Yusuke Shiode, Mio Hosokawa, Masayuki Hirano, Mika Hosogi, Atsushi Fujiwara, Kazuhisa Miyamoto, Fumio Shiraga

**Affiliations:** Department of Ophthalmology, Okayama University Graduate School of Medicine, Dentistry and Pharmaceutical Sciences, 2-5-1 Shikata-cho Kita-ku, Okayama, Japan; Department of Ophthalmology, Sumitomo-Bessi Hospital, Niihama, Japan

**Keywords:** Traumatic submacular hemorrhage, Vitrectomy, Subretinal injection, Recombinant tissue plasminogen activator, Air tamponade, Choroidal rupture

## Abstract

**Background:**

The natural course of submacular hemorrhage resulting from traumatic choroidal rupture generally has a poor outcome unless treated. The intravitreal injection of gas only or gas with recombinant tissue plasminogen activator (rt-PA) has been reported to be effective, but has also been reported to induce severe complications such as retinal detachment and vitreous hemorrhage. Recently, we reported a safe and effective procedure for treating submacular hemorrhage due to polypoidal choroidal vasculopathy (PCV) with a low dose of rt-PA. Here we report the application of this procedure to a case of traumatic submacular hemorrhage in a 13-year-old boy, which achieved a good visual outcome.

**Case presentation:**

A 13-year-old Japanese boy presented with a thick submacular hemorrhage in his left eye as a result of blunt trauma from being hit by a sinker. Best-corrected visual acuity (BCVA) was assessed as only able to perceive hand motions. We carried out a vitrectomy, subretinal injection of 4,000 IU rt-PA (6.9 μg) and air tamponade. The day after surgery, most of the submacular hemorrhage had moved to the inferior periphery. One month after the surgery, we observed cataract formation, thin remnants of the submacular hemorrhage and juxtafoveal choroidal rupture. We carried out cataract surgery and injected bevacizumab intravitreally to prevent the development of choroidal neovascularization. Two months after the second surgery, the submacular hemorrhage had totally disappeared and the patient had a BCVA of 20/40.

**Conclusion:**

Vitrectomy, subretinal injection of rt-PA, and intravitreal air tamponade may be a promising strategy for treating traumatic submacular hemorrhage in young patients.

## Background

Ocular blunt trauma can rupture the choroid and result in massive submacular hemorrhage [1]. The outcomes for untreated traumatic submacular hemorrhages have been reported to be poor because of the resulting hematotoxicity, the development of choroidal neovascularization arising from choroidal rupture, and subsequent fibrotic degeneration of the retina and choroid [2, 3].

For patients to recover good visual acuity, surgical intervention should be considered. To date, traumatic submacular hemorrhage was often treated with the intravitreal injection of gas only or gas with recombinant tissue plasminogen activator (rt-PA) without carrying out a vitrectomy [4–7]. However, injecting gas into non-vitrectomized eyes has been reported to induce severe complications, such as retinal detachment and vitreous hemorrhage [4, 8].

Recently, we have reported that vitrectomy, subretinal injection of rt-PA and air tamponade were effective in treating submacular hemorrhage due to PCV [8]. This procedure assured the safe displacement of submacular hemorrhages, using a low dose of rt-PA. We therefore applied the same procedure to a traumatic submacular hemorrhage in a 13-year-old boy and achieved a good visual outcome.

## Case presentation

**Fig. 1:**
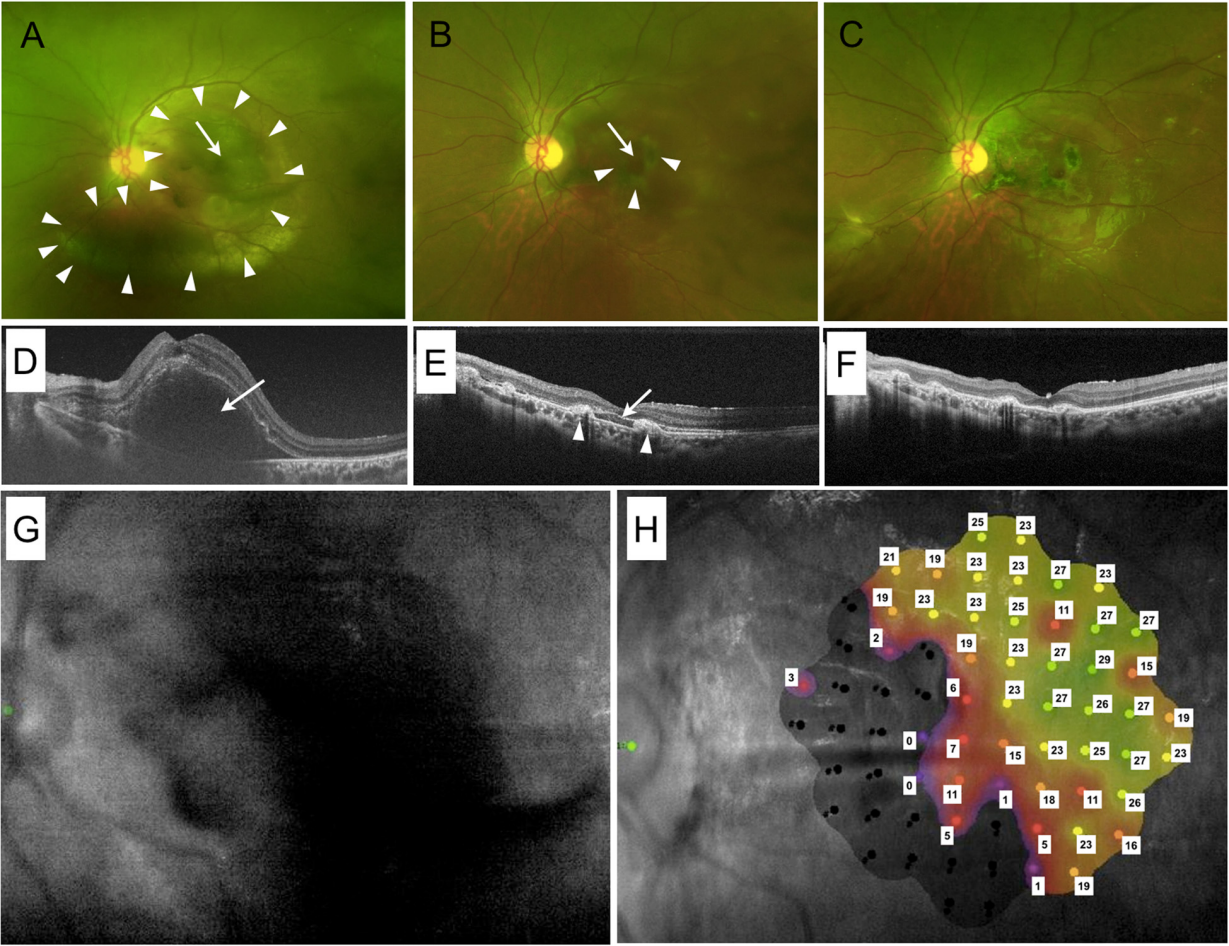
Successful displacement of a traumatic submacular hemorrhage in the left eye of a 13-year-old boy. Fundus examinations (**a**) before surgery, when the best-corrected visual acuity (BCVA) was hand motions only, showing a subretinal hemorrhage extending from the posterior pole to the inferior midperiphery (arrow indicates the thick submacular hemorrhage and arrow-heads outline its area), (**b**) one month after vitrectomy, when the BCVA was 20/1000, showing the remaining thin submacular hemorrhage and juxtafoveal choroidal ruptures (arrow indicates the submacular hemorrhage and arrow-heads indicate juxtafoveal choroidal ruptures), and (**c**) two months after the second operation, when the BCVA of the left eye had improved to 20/40, showing the submacular hemorrhage had totally disappeared. Optical coherence tomography (**d**) before surgery (arrow indicates the thick submacular hemorrhage), (**e**) one month after vitrectomy showing the remaining thin submacular hemorrhage and juxtafoveal choroidal ruptures (arrow indicates the submacular hemorrhage and arrow-heads indicate juxtafoveal choroidal ruptures), and (**f**) two months after the second operation, showing the submacular hemorrhage had totally disappeared. Retinal sensitivity measured with a microperimeter (MAIA) (**g**) could not be detected before surgery, but (**h**) showed a marked improvement two months after the second operation. The numbers in H indicate retinal sensitivities (dB)

An otherwise healthy 13-year-old Japanese boy presented with reduced central vision in his left eye, 3 days after suffering a blunt trauma from being hit by a fishing sinker. Assessment of BCVA showed only the ability to perceive hand motions in the left eye compared to 20/13.3 in the right eye. The intraocular pressure was 8 mmHg in both eyes. The anterior segment of the left eye showed hyphema and laceration of the bulbar conjunctiva. No traumatic cataract or rupture of the globe was seen. Fundus examination of the left eye showed a subretinal hemorrhage extending from the posterior pole to the inferior midperiphery and a slight vitreous hemorrhage (Fig. 1a). No retinal tear or retinal detachment was seen. In contrast, the right eye appeared normal. Swept-source optical coherence tomography (OCT; DRI OCT-1 Atlantis, Topcon Medical Systems, Tokyo, Japan) showed the presence of a thick submacular hemorrhage in the left eye (Fig. 1d). Attempts to measure the retinal sensitivity using a microperimeter (MAIA; CenterVue, Padova, Italy) failed, as it was below the detection limits of the instrument (Fig. 1g). We therefore diagnosed submacular hemorrhage in the left eye, due to choroidal rupture following blunt trauma.

Two days after the initial visit, we carried out anterior chamber lavage, vitrectomy, subretinal injection of rt-PA (GRTPA®, Mitsubishi Tanabe Pharma Corporation, Japan) and air tamponade on the left eye under general anesthesia. This operation was performed using the methods reported by Kimura *et al.* [8]. Briefly, after performing a 25-gauge micro-incision vitrectomy, 4,000 IU rt-PA in 0.1 ml was injected subretinally using a 38-gauge subretinal infusion needle (MedOne, Sarasota, FL) to liquefy the submacular hemorrhage. Before completing the operation, fluid-air exchange was performed. The patients remained facedown for 3 days after surgery to displace the submacular hemorrhage. The day after the operation, most of the submacular hemorrhage had moved to the inferior periphery.

One month after the operation, the BCVA of left eye was 20/1000 and we observed cataract formation, thin remnants of the submacular hemorrhage and juxtafoveal choroidal rupture (Fig. 1b and e). We then carried out phacoemulsification and aspiration, intraocular lens implantation and intravitreal injection of 0.05 ml bevacizumab (Avastin®, Hoffmann-la Roche, Basel, Switzerland) in the left eye under general anesthesia. Two months after the second operation, the submacular hemorrhage had totally disappeared (Fig. 1c and f) and the BCVA in the left eye had improved to 20/40. As shown in Fig. 1h, the retinal sensitivity had markedly improved.

## Conclusions

We successfully displaced a traumatic submacular hemorrhage in the eye of a 13-year-old boy using vitrectomy, the subretinal injection of 4,000 IU rt-PA, and air tamponade. Previously, Hillenkamp *et al.* reported a case of traumatic submacular hemorrhage in which they injected 10–20 μg rt-PA subretinally [9]. The dose of rt-PA we used was 35–69 % of the dose they had used and 14–28 % of the rt-PA dose which has generally been injected subretinally to displace submacular hemorrhage due to age related macular degeneration or macroaneurysm [9–12]. Since rt-PA has been reported to cause retinal toxicity, such as a decreased amplitude in electroretinography and atrophy of photoreceptors [3, 12–14], it is important to carefully consider the dose of rt-PA used. Although the post-operative observation period for this case has only been 3 months, no chorioretinal atrophy or reduced retinal sensitivity has so far been observed. Longer term follow-up will be needed to monitor any retinal toxicity due to rt-PA.

One month after surgery, we observed a juxtafoveal choroidal rupture and a residual submacular hemorrhage. We then injected bevacizumab intravitreally, to prevent the development of choroidal neovascularization and accelerate absorption of the submacular hemorrhage. Choroidal rupture is known to be a major risk factor for the development of choroidal neovascularization, with a reported rate of 11.0–37.5 % [3, 13–15]. In this case, we concerned about development of choroidal neovascularization, which was likely to directly affect visual acuity, because the choroidal rupture was located in the juxtafoveal region. Abdul-Salim *et al.* reported a case in which the intravitreal injection of bevacizumab contributed to the absorption of a submacular hemorrhage due to traumatic choroidal rupture [15]. They speculated that the anti-inflammatory effect of the anti-VEGF drug promoted the absorption of the submacular hemorrhage. Although choroidal neovascularization has not been seen so far in our patient, longer follow-up is needed to monitor for its development.

At present, the treatment for submacular hemorrhage can be broadly divided into “nonvitrectomizing techniques” and “vitrectomizing techniques.” Both approaches have been combined with intravitreal injection of rt-PA or anti-VEGF or gas or a combination of these. Especially in young patient, traumatic submacular hemorrhage may often take a wait-and-see approach or nonvitrectomizing techniques. Because vitrectomizing techniques for young patient can cause severe complications such as proliferative vitreoretinopathy or rhegmatogenous retinal detachment. In the past, there are a few reports for young traumatic submacular hemorrhage. These procedures are intravitreal injection of gas, ranivizmab, or gas and rt-PA [6, 7, 15]. In this case, we chose vitrectomizing technique even though the patient is 13 years old young boy. Because the submacular hemorrhage is very large and thick, so we think we can not remove this submacular hemorrhage by nonvitrectomizing techniques only. Furthermore, subretinal injection of rt-PA is efficient for adult massive submacular hemorrhage due to PCV [8], we selected the same procedure for this case. Fortunately, submacular hemorrhage moved effectively out of fovea and no severe complication occered except cataract formation. We must take care when we are taking vitrectomizing technique for young patient.

In summary, vitrectomy, subretinal injection of rt-PA and air tamponade may be a promising strategy for treating traumatic submacular hemorrhage in young patients. However, this report describes only a single case, with a relatively short follow-up period so far, and randomized controlled clinical studies involving a larger number of patients will be needed to determine the impact of this surgical procedure on the management of traumatic subretinal hemorrhage.

### Consent

Written informed consent was obtained from the patient for publication of this case report and accompanying images. A copy of the written consent is available for review by the Editor-in-Chief of this journal.

### Parental consent

Written Parental informed consent was obtained from the parents' patient for publication of this case report and any accompanying images. A copy of the written Parental consent is available for review by the Editor-in-Chief of this journal.

## References

[1] Hochman MA, Seery CM, Zarbin MA (1997). Pathophysiology and management of subretinal hemorrhage. Surv Ophthalmol.

[2] Glatt H, Machemer R (1982). Experimental subretinal hemorrhage in rabbits. Am J Ophthalmol.

[3] Ament CS, Zacks DN, Lane AM, Krzystolik M, D'Amico DJ, Mukai S, Young LH, Loewenstein J, Arroyo J, Miller JW (2006). Predictors of visual outcome and choroidal neovascular membrane formation after traumatic choroidal rupture. Arch Ophthalmol.

[4] Ohji M, Saito Y, Hayashi A, Lewis JM, Tano Y (1998). Pneumatic displacement of subretinal hemorrhage without tissue plasminogen activator. Arch Ophthalmol.

[5] Yang P-M, Kuo H-K, Kao M-L, Chen Y-J, Tsai H-H (2005). Pneumatic displacement of a dense submacular hemorrhage with or without tissue plasminogen activator. Chang Gung Med J.

[6] Holland D, Wiechens B (2004). Intravitreal r-TPA and gas injection in traumatic submacular hemorrhage. Ophthalmologica.

[7] Goldman DR, Vora RA, Reichel E (2014). Traumatic choroidal rupture with submacular hemorrhage treated with pneumatic displacement. Retina.

[8] Kimura S, Morizane Y, Hosokawa M, Shiode Y, Kawata T, Doi S, Matoba R, Hosogi M, Fujiwara A, Inoue Y, Shiraga F (2014). Submacular hemorrhage in polypoidal choroidal vasculopathy treated by vitrectomy and subretinal tissue plasminogen activator. Am J Ophthalmol.

[9] Hillenkamp J, Surguch V, Framme C, Gabel V-P, Sachs HG (2010). Management of submacular hemorrhage with intravitreal versus subretinal injection of recombinant tissue plasminogen activator. Graefes Arch Clin Exp Ophthalmol.

[10] Fine HF, Iranmanesh R, Del Priore LV, Barile GR, Chang LK, Chang S, Schiff WM (2010). Surgical outcomes after massive subretinal hemorrhage secondary to age-related macular degeneration. Retina.

[11] Haupert CL, McCuen BW, Jaffe GJ, Steuer ER, Cox TA, Toth CA, Fekrat S, Postel EA (2001). Pars plana vitrectomy, subretinal injection of tissue plasminogen activator, and fluid-gas exchange for displacement of thick submacular hemorrhage in age-related macular degeneration. Am J Ophthalmol.

[12] Johnson MW, Olsen KR, Hernandez E, Irvine WD, Johnson RN (1990). Retinal toxicity of recombinant tissue plasminogen activator in the rabbit. Arch Ophthalmol.

[13] Secrétan M, Sickenberg M, Zografos L, Piguet B (1998). Morphometric characteristics of traumatic choroidal ruptures associated with neovascularization. Retina.

[14] Wyszynski RE, Grossniklaus HE, Frank KE (1988). Indirect choroidal rupture secondary to blunt ocular trauma. A review of eight eyes. Retina.

[15] Abdul-Salim I, Embong Z, Khairy-Shamel S-T, Raja-Azmi M-N (2013). Intravitreal ranibizumab in treating extensive traumatic submacular hemorrhage. Clin Ophthalmol.

